# Schwannomatosis of Cervical Vagus Nerve

**DOI:** 10.1155/2016/8020919

**Published:** 2016-10-11

**Authors:** Faheem Ahmed Abdulla, M. P. Sasi

**Affiliations:** Department of General Surgery, Government Medical College, Kozhikode, Kerala, India

## Abstract

Cervical vagal schwannoma is a rare entity among lesions presenting as a neck mass. They are usually slow-growing benign lesions closely associated with the vagus nerve. They are usually solitary and asymptomatic. Multiple schwannomas occurring in patients without neurofibromatosis (NF) are rare and have recently been referred to as schwannomatosis. Here, we present a case of a neck mass that had imaging features suggestive of vagal schwannoma and was operated upon. Intraoperatively, it was discovered to be a case of multiple vagal cervical schwannoma, all directly related to the right vagus nerve, and could be resected from the nerve in toto preserving the function of the vagus nerve. Final HPR confirmed our pre-op suspicion of vagal schwannomatosis.

## 1. Introduction

Schwannoma is defined as a benign tumor of neural origin derived from the benign nerve sheath composed of Schwann cells, which normally produce the insulating myelin sheath covering the peripheral nerves [1]. Although usually synonymous with acoustic neuroma, schwannoma may also arise from other nerves other than the vestibulocochlear nerve, albeit rarely. Schwannoma of the vagus nerve is a rare entity, seen in only a handful of cases [2]. Of the reported cases, most have been seen among middle aged men as an asymptomatic neck swelling [1]. Intraoperatively, they have been seen as a solitary swelling arising from the cervical vagus nerve, even though mediastinal origin has also been seen. Schwannomatosis is a term relatively recently introduced that defines a condition of multiple schwannomas related to a cranial nerve, spinal nerve root, or a peripheral nerve [3]. Multiple schwannomas are usually seen in cases of neurofibromatosis 2 but rarely seen otherwise. Other differentials to be considered include paragangliomas, cervical lymphadenopathy, branchial cyst, and malignant neck swellings [4]. As there are no specific imaging or histological features, a great deal of clinical suspicion is needed to make an accurate pre-op diagnosis [1]. We hereby present a rare case of schwannomatosis of the cervical vagus nerve not otherwise associated with neurofibromatosis 2 (NF-2) [5].

## 2. Case Report

**Figure 1:**
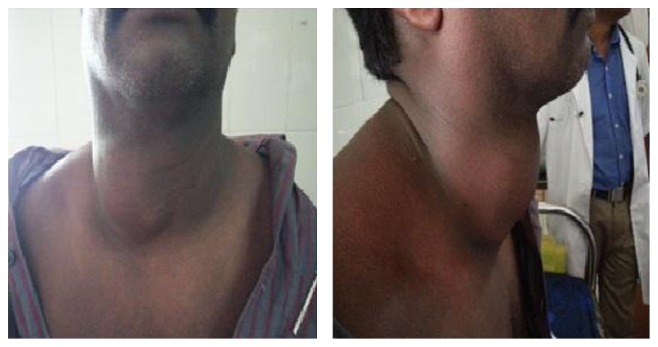
Profile of the patient showing the swelling from front and side.

A 40-year-old male patient presented to our department with complaints of right sided neck swelling that had gradually progressed in size over the past 5 yrs. He does not give any history of hoarseness of voice, recent cough, or light headedness. He complains of difficulty swallowing and also in neck movements. On examination of the patient, a firm, immobile, nontender swelling of size 10 × 10 cm was found, which involved the right side of neck. It was seen extending from behind the right sternocleidomastoid muscle to just across the midline of neck. Vertically, it extended from the hyoid bone to the sternal end of right clavicle. It was nonpulsatile and no murmur was audible. Carotid artery was displaced clinically by the mass. Examination revealed all cranial nerves to be within normal limits. No other swellings were palpable in the neck (Figure 1).

**Figure 2:**
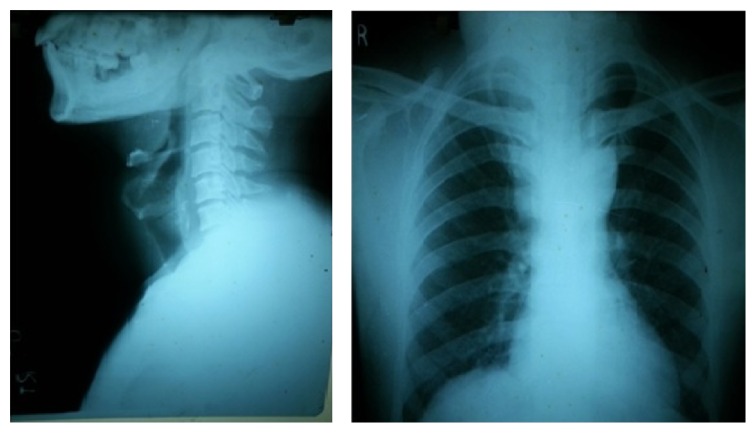
Cervical and thoracic X-rays to look for any obstructive signs.

Cervical spine and thoracic X-rays were taken to see for tracheal obstruction and mediastinal shadow as well as pre-op planning. Cervical X-ray did not show any tracheal narrowing while there was a slight suspicion of mediastinal widening (Figure 2).

**Figure 3:**
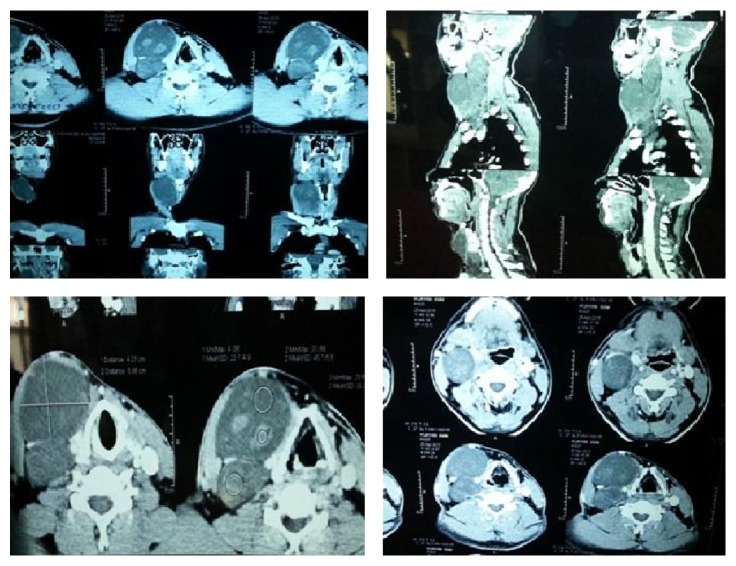
CECT images showing the lesion in the neck, extending to submandibular area and mediastinum.

For further characterisation of the swelling, a contrast enhanced computed tomography was done which showed presence of fairly well-defined hypodense mass lesions in the right side of neck extending to upper mediastinum. The largest lesion was approximately 4.2 × 5.4 cm in anteroposterior and transverse dimensions. The craniocaudal extent of the lesions was approx. 17.8 cm. There was poor contrast enhancement of the lesions with few mildly enhancing internal components. The upper part of the mass was abutting the pterygoid muscles and the C2 vertebrae. The lower level of the lesion was at the level of the carina. The masses in the neck were mainly involving the right carotid space displacing and separating the carotid arteries and the internal jugular vein suggestive of a neurogenic tumor. The lesions were also displacing the right SCM muscle and right submandibular gland. The thyroid and cricoid cartilages, hypopharynx, larynx, and trachea were displaced to the left without obvious evidence of erosion or infiltration. In the mediastinum, the lesion was seen to displace the azygous vein, right brachiocephalic vein, and superior vena cava and abutting the aortic arch and right main bronchus. The thyroid gland appeared normal. There was no obvious cervical lymph node enlargement, no intracranial extension, nor any obvious bone or cartilage erosion in the area of study. The present CECT was suggestive of a neurogenic tumor probably from the right vagus nerve (Figure 3).

**Figure 4:**
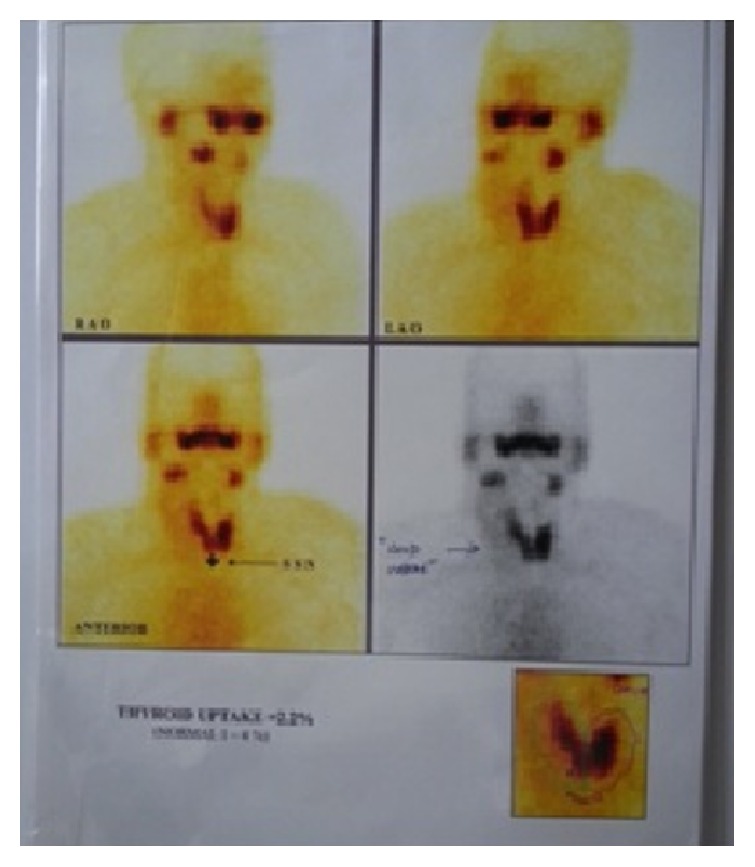
Tc99m scintigraphy showing normal uptake in the thyroid gland, a cold nodule in the right lobe, and normal activity in the salivary gland.

A Tc99m thyroid scintigraphy was done from another institution before presentation which showed presence of normal thyroid gland with normal uptake, but for the presence of a cold nodule in the right lobe. Under USG guidance, an FNA was done from the cold nodule in the right lobe of thyroid. FNAC result revealed a benign colloid nodule. As malignancy was ruled out, no further action was taken on the thyroid gland. The swelling in question was not evident on the scintigraphy (Figure 4).

An FNA was taken from the swelling in question, which was inconclusive.

Further routine workup was done which included basic blood and renal and thyroid workup which were within normal limits, and patient was prepared for surgery.

**Figure 5:**
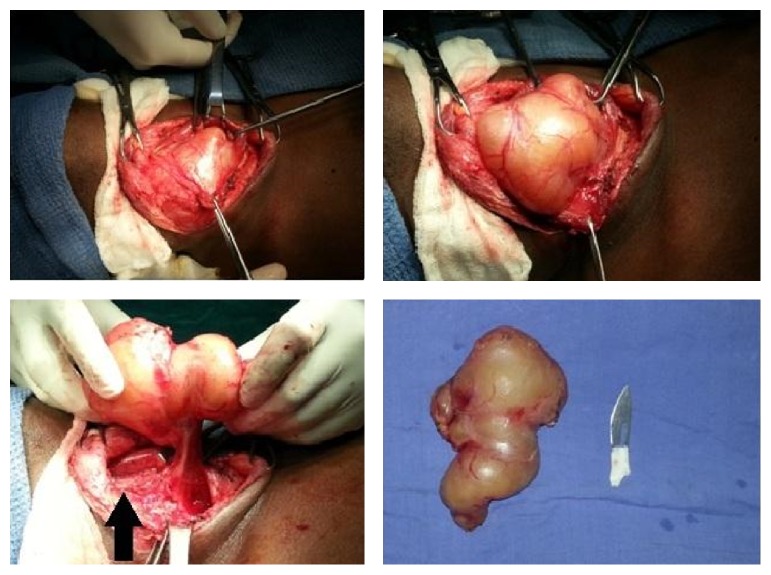
The first specimen seen related to vagus nerve (black arrow) and resected specimen measuring 12 × 7 cm.

Under general anesthesia, a collar-line transverse incision was made 1 cm below the cricoid cartilage and extending along the anterior border of the right sternocleidomastoid. After raising subplatysmal flaps, the strap muscles were separated, and the lesion was visualised. A yellowish mass lesion was visualised extending beyond the midline which appeared well-defined and well-encapsulated. It was dissected from the adjacent tissue and found to have posterior attachment to the ipsilateral vagus nerve. The vagus nerve was splayed over its surface and carefully dissected off the swelling, thereby preserving the whole of the nerve. There was an episode of bradycardia during this manoeuvre, but it was transient and the rest of the procedure was uneventful (Figure 5).

**Figure 6:**
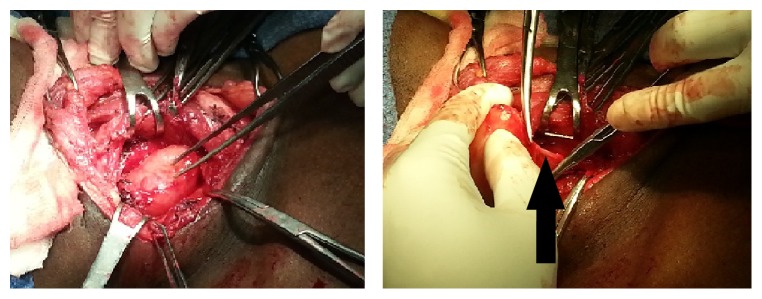
The rest of the lesions were also similarly resected preserving the vagus nerve (black arrow).

After successful removal of the lesion, there were multiple other similar swellings seen discontinuous from the first, along the vagus nerve, proximal and distal to the first swelling. They were also dissected away from the vagus nerve and removed in toto. Lesions involving the submandibular area were also resected. The mediastinal lesion seen on imaging was removed along with the first specimen via the same incision itself. Hemostasis was ensured and the wound was closed in layers after placing a suction drain. The patient had an uneventful postoperative period and was discharged on the 5th post-op day. There was no obvious evidence of vagus nerve injury, no hoarseness, or cough (Figure 6).

**Figure 7:**
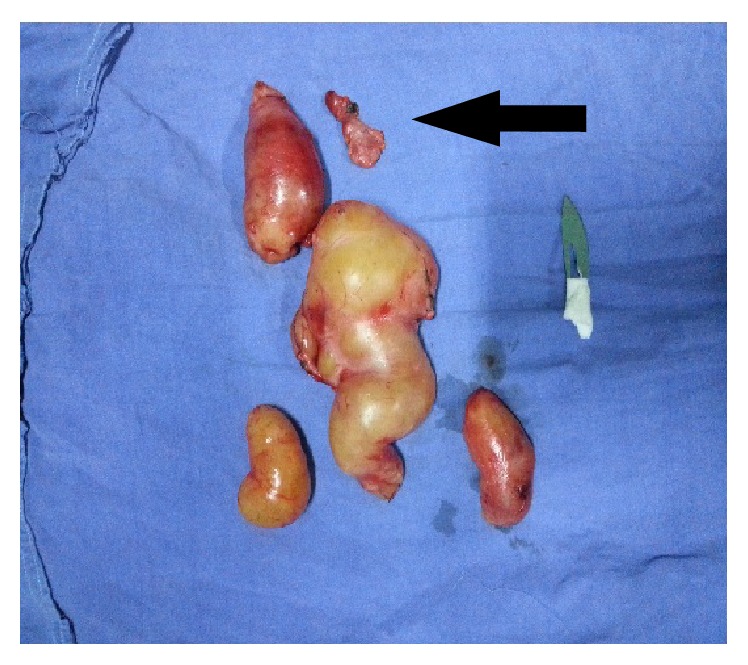
The entire resected specimens with one lymph node (black arrow).

In total, there were 5 specimens, of which one was a lymph node from level 2 that was also excised (Figure 7).

Final histopathology report showed the presence of schwannoma with degeneration, composed of focal hypercellular (Anthony A) areas and hypocellular areas (Anthony B). Immunohistochemical examination of tumor cells showed strong S-100 positivity. Therefore, a final diagnosis of vagal nerve schwannomatosis was obtained. The lymph node was histologically unremarkable.

## 3. Discussion

Most of the extracranial schwannomas are present in the head and neck area; the most commonly affected regions are the temporal bone, lateral neck, and paranasal sinuses [6]. Schwannomas developing from cranial nerves are mostly from vestibulocochlear nerve; rarely the glossopharyngeal, accessory, and hypoglossal nerves may also be affected. The vagus nerve is involved in approx. 10% of cases [6].

Vagal schwannoma is usually seen in middle aged men but may also occur in females. Although an asymptomatic presentation is common, they can also present with hoarseness and cough. Cough elicited by pressure over the swelling is a sign used by some to diagnose vagal schwannoma [7].

Diagnosing vagal schwannoma preoperatively may be an issue, since there are no classic imaging or cytological criteria to diagnose them [8]. In a study based on extracranial schwannoma in head and neck, it was found that only 6% of patients can be diagnosed preoperatively on the basis of clinical findings, CT and MRI scans, and FNA [2]. In another related study it was found that the cytological diagnosis of schwannoma was only definitive in 20% of cases, based on the characteristic features [6]. The classical histological picture is one composed of Antony A bodies which signify compact spindle cells and Antony B bodies which compromise looser arranged Schwann cells [9].

Adequate treatment includes complete removal of lesions safeguarding the nerve involved, if possible. In vagal schwannoma, priority should be given to preserving the nerve due to the morbidity associated with damage to vagus nerve, but in some cases, resection of a portion of the nerve with end to end anastomosis has been done with mixed results [8]. The malignant potential of vagal schwannoma and its risk of recurrence are unknown. In most studies, a low risk of recurrence, if any, was noted. Hence complete surgical excision will suffice [2, 6].

Schwannomas have been commonly seen associated with neurofibromatosis 1 and neurofibromatosis 2, but mostly NF 2. When multiple schwannomas are present in the absence of other stigmata of NF, then it can be defined as schwannomatosis [10].

Schwannomatosis was first described in 1973 as neurofibromatosis type 3 [9]. Along the years, many papers have been published noting further cases of schwannomatosis without features of NF, thereby signalling a new entity. As per Jacoby et al., the presence of two or more schwannomas in the absence of radiological evidence of vestibular lesions in patients older than 18 years is definitely indicative of schwannomatosis [11]. According to Baser et al., MRI should be used to rule out presence of vestibular schwannoma and also any presence of NF mutations should also be ruled out [12].

The reported incidence of preoperative vocal cord paralysis is about 12%, but hoarseness is almost always present following surgery. Therefore, vocal cord status should be assessed prior to surgery [13]. Since vagal schwannomas are almost always benign in nature, a conservative approach should always be considered in first instance when the integrity of the nerve is in question [14].

## 4. Conclusion

Vagal schwannomas are rare benign lesions of the head and neck. They present insidiously and are usually asymptomatic. With a degree of clinical suspicion and proper imaging studies, a diagnosis can be made most of the time. Surgical technique is crucial since preservation of the integrity of the vagus nerve is of paramount importance. Considering the rarity of schwannomatosis of the vagus nerve, adequate care should be given before, during and after surgical treatment.
